# Cardiorespiratory Fitness Normative Values in Latin-American Adolescents: Role of Fatness Parameters

**DOI:** 10.3390/ijerph16203889

**Published:** 2019-10-14

**Authors:** Robinson Ramírez-Vélez, Antonio García-Hermoso, Alicia María Alonso-Martínez, César Agostinis-Sobrinho, Jorge Enrique Correa-Bautista, Héctor Reynaldo Triana-Reina, Mikel Izquierdo

**Affiliations:** 1 Department of Health Sciences, Public University of Navarra, 31008 Pamplona, Navarra, Spain; aliciamaria.alonso@unavarra.es (A.M.A.-M.); correab.jorge@gmail.com (J.E.C.-B.); mikel.izquierdo@gmail.com (M.I.); 2 Navarrabiomed-Universidad Pública de Navarra (UPNA)-Complejo Hospitalario de Navarra (CHN), Instituto de Investigación Sanitaria de Navarra (IdiSNA), 31008 Pamplona, Navarra, Spain; antonio.garciah@unavarra.es; 3 Laboratorio de Ciencias de la Actividad Física, el Deporte y la Salud, Universidad de Santiago de Chile, USACH, Santiago 9160030, Chile; 4 Research Centre in Physical Activity, Health and Leisure (CIAFEL), Faculty of Sport, University of Porto, 4200-450 Porto, Portugal; cesaragostinis@hotmail.com; 5 Faculty of Health and Sciences, Klaipeda University, 92294 Klaipeda, Lithuania; 6 GICAEDS Group, Faculty of Physical Culture, Sport and Recreation, Universidad Santo Tomás, 110311 Bogotá, Colombia; hectortriana@usantotomas.edu.co; 7 CIBER of Frailty and Healthy Aging (CIBERFES), Instituto de Salud Carlos III, 28001 Madrid, Spain

**Keywords:** aerobic fitness, physical performance, obesity, adolescents, prevention, cardiovascular risk factors

## Abstract

The aim of this study was to provide percentile values for a cardiorespiratory fitness (CRF) field test for Latin-American adolescents (34,461 girls and 38,044 boys) aged 13 to 15 years. The role of fatness parameters on the CRF level across age groups was also examined, with a focus on non-obese (healthy) and obese groups. CRF was assessed using the 20-meter shuttle run test protocol. Anthropometric parameters were measured using body mass index z-score (body mass index (BMI) z-score), BMI, waist circumference (WC), and waist-to-height ratio (WHtR). Participants were categorized according to the BMI z-score, WC, and WHtR international cut-off points as healthy and obese. Age- and sex-specific reference tables for the 3rd, 10th, 25th, 50th, 75th, 90th, and 97th centile scores were calculated using Cole’s lambda, mu, and sigma method. The prevalence of obesity according to the BMI z-score, WC, and WHtR was 9.6%, 11.2%, and 15.0%, respectively. Across all age and sex groups, a negative association was found between relative peak oxygen uptake ($\dot{V}$O_2_peak) and BMI, WC, and WHtR. In boys and girls there were higher levels of performance across all age groups, with most apparent gains between the ages of 13 and 14 years old. Overall, participants categorized in the healthy group had shown to have significantly higher $\dot{V}$O_2_peak than their obese counterparts (*p* < 0.001; Cohen’s *d* > 1.0). In conclusion, our study provides age- and sex-specific reference values for CRF ($\dot{V}$O_2_peak, mL·kg^−1^·min^−1^). The anthropometric parameters were inversely associated with CRF in all ages in both sexes. The obese group had worse CRF than their healthy counterparts independent of anthropometric parameters used to determine obesity.

## 1. Introduction

Increasing attention is being given to the importance of cardiorespiratory fitness (CRF) levels, for decreasing chronic diseases, promoting overall cardiovascular and general health, and improving quality of life [1]. Mounting evidence over the past years has firmly established that low CRF is strongly and independently associated with a high risk of cardiovascular disease [2], all-cause mortality [3], diabetes [4], mental health [5], stroke [6], various types of cancers [7], and many other risk factors and comorbidities.

In children and adolescents, peak oxygen uptake ($\dot{V}$O_2_peak) is the most researched physiological variable in pediatric exercise physiology for its association with cardiometabolic risk factors [8,9] and other health-related variables [10]. The current literature indicates that there has been a substantial decline in CRF in high-income and upper middle-income countries in the last decades [11]. Moreover, there is a gap on CRF data for low-income and middle-income countries, underscoring the need to improve the monitoring of CRF across populations because temporal declines are indicative of declines in population health [11].

CRF can be objectively assessed by lab-based, but the need of expensive equipment limits its use in epidemiological levels, such as in school settings. To help address these limitations, field tests might be an alternative for estimation/prediction CRF described as $\dot{V}$O_2_peak in children and youth. The 20-meter shuttle run test (20mSRT) is the most widely used field-based estimation of CRF in youth worldwide [12]. In addition, it is has been demonstrated to be an suitable tool (feasible, valid, and reliable) for population-based surveillance and monitoring, due to its low cost, flexibility with testing locations, and its ability to test multiple individuals simultaneously [13].

Two recent studies [12,14] have presented international criterion-referenced standards regarding CRF for children and adolescents. However, it is noteworthy that both observational studies have identified populations in several high-income countries, and to the best of our knowledge, there are not any studies among a large sample of south American children or adolescents. In addition, there is a need to track most accurately trends on CRF in youth, in large-scale, harmonized national health and fitness surveys, especially among low-income and middle-income countries [13]. In this sense, and from a public health perspective, providing age-, and sex-specific percentiles for CRF field tests for Latin-American adolescents should support possible interventions or prevention programs on a broader range aiming to improve the health status.

In addition to the decreased CRF levels in Latin American adolescents, the prevalence of overweight and obesity among this population has widely increased, making it one of the most common chronic disorders in this age group and in adulthood [15]. Overall, increased fatness status has a negative influence on CRF levels [16] besides, there are age and sex specific differences accordingly to weight status and CRF during adolescence [12,16].

However, given the role of age, sex, and obesity on CRF levels, the aim of this study was to provide percentile values for CRF field tests for Latin-American adolescents (38,044 boys and 34,461 girls) aged 13 to 15 years old. In addition, the influence of fatness parameters on the CRF level across age groups was also examined, with a focus on healthy and obese groups.

## 2. Materials and Methods 

### 2.1. Design Study and Subjects 

The current study is based on pooled analyses data obtained from two separate and independent samples: Chile (*n* = 47,715; SIMCE, in Spanish *Sistema de Medición de la Calidad de la Educación*; in English *System for the Assessment of Educational Quality*) and Colombia (*n* = 24,790; “Prueba SER Survey and Fuprecol Project”). Detailed information about the objectives, design, and protocol has been published elsewhere [17]. Briefly, “the SIMCE” study was based on a representative sample of Chilean 8th grade students (mean age=14.0 ± 0.6 years old), and maximal effort exercise was not contraindicated [18]. The sample was stratified by 15 regions and three school categories. The National Physical Education Survey was authorized under the Chilean Sports Law number 19.712, Article 5 [18]. 

The second sample was taken from the “Prueba SER survey and Fuprecol Project”. These were cross-sectional surveys of ninth grade students recruited from public and private schools in all 20 municipalities within the District Capital of Bogota (Cundinamarca Department, Andean Region of Colombia). Further details can be obtained from the website (available from: https://www.educacionbogota.edu.co/archivos/Temas%20estrategicos/Documentos/Resultados_PruebasSER-Bienestar_Fisico_Ciudadania_y_Convivencia.pdf). The study protocols were approved by the Universidad de Santiago de Chile (Chile) by th Institutional Review Board and the Review Committee for Research Involving Human Subjects at Bogotá’s District Secretary of Education (ID Convenio N CDP 3381, Project N 893 “Pensar en Educación” date 2 October 2014, and Fuprecol Project Code N CEI-ABN026–000262, date 27 September 2013). 

### 2.2. Procedures

Weight and height were measured following standard procedures by the same trained investigator in each study country. Body fatness parameters were estimated using body mass index (BMI, kg/m^2^) and waist circumference (WC) information. BMI and age- and sex-specific BMI z-scores according to the World Health Organization reference standards were calculated, with obesity defined as ≥ 2 SD [19]. WC and height were used to calculate waist-to-height ratio (WHtR), with obesity defined as ≥ 0.50 according to previous reports [20,21]. The 75th percentile of WC according to age and sex were determined based on data collected from the de Ferranti et al. [22]. After defining obesity using BMI z-score, WHtR and 75th percentile of WC, a new variable was generated from the combination of both. Students were classified as obese simultaneously (BMI z-score + WC or BMI + WHtR). 

CRF was assessed using the 20-meter shuttle run test (20mSRT) protocol [23,24]. Results were recorded to the nearest stage (minute) completed with relative $\dot{V}$O_2_peak (in mL·kg^−1^·min^−1^) estimated using the Léger equation [24]. The 20mSRT demonstrates strong test-retest reliability and moderate-to-strong validity which suggests that the 20mSRT is a surrogate measure to estimating/predicting of $\dot{V}$O_2_peak [25]. 

### 2.3. Statistical Analyses

Participants were divided into three age groups: 13, 14 and 15 years old. Age- and sex-specific means, SD, frequency and percentage were calculated. Sex differences were compared by two-way analysis of variance, as well as the differences between anthropometric parameters, and the subgroup means were compared using the Bonferroni test. The categorical values were compared by Chi-squared test (χ^2^-test). Percentile values (3rd, 10th, 25th, 50th, 75th, 90th and 97th) were fitted to the data using Cole’s transformation (L for skewness), median (M), and coefficient of variation (S) method [26]. Linear regression (standardized regression coefficient with 95% confidence interval) were calculated to quantify the relationship between $\dot{V}$O_2_peak according to equation of Léger et al. [24] and fatness parameters (BMI, WC and WHtR), adjusted by country. We observed significant interactions by sex (*p* < 0.01), hence the linear regression analyses were performed for boys and girls in an independent manner. The Cohen’s *d* (effect size) statistic was calculated by determining the difference between two groups (healthy and obesity), thus, Cohen’s *d* = *M*1 − *M*2/σ_pooled_, where σ_pooled_ = √[(σ 1^2^ + σ 2^2^)/2]. The magnitude was interpreted by using small (0.20 to <0.50), moderate (0.50 to <0.80), and large (≥0.80) values. Statistical analyses were carried out using the SPSS software® 24 version for Windows (IBM-SPSS Inc., Chicago, IL, USA). Smoothed age and gender-specific for all percentiles were constructed with the use Lambda-mu-sigma Chart Maker Pro program software® (version 2.54, Medical Research Council, Cambridge, UK). *p* value <  0.05 was considered to be statistically significant.

## 3. Results

We included participants who completed the fatness and $\dot{V}$O_2_peak with non-missing values for all tests (n = 86 were excluded). The final sample comprised 72,505 adolescents aged 14.02 (95%CI 14.02−14.03) years old. Means (standard deviation, SD) for the full sample were: BMI, 21.65 (3.53) kg/m^2^; WC, 71.12 (8.48) cm; WHtR, 0.44 (0.05); and CRF, 42.49 (6.53) mL·kg^−1^·min^−1^ using the Léger et al. equation [24]. Boys had significantly higher values than girls in weight, height, WC, and $\dot{V}$O_2_peak, except in BMI and BMI z-score. The prevalence of obesity according to the BMI z-score was 9.6%, and the prevalence of central obesity according to the >75th percentile for age and sex was 11.2%, and WHtR (≥0.50) was 15.0%, (Table 1).

As expected, across all age and sex groups, a significant negative association was found between $\dot{V}$O_2_peak and BMI, WC, and WHtR (Table 2). 

Table 3 shows smoothed centile scores (the 3rd, 10th, 25th, 50th, 75th, 90th, and 97th) and Cole’s transformation (L for skewness), median (M), and coefficient of variation (S) values for percentiles for the $\dot{V}$O_2_peak, across all age and sex groups. It can be observed that CRF performance in boys was generally more homogeneous than in girls. In girls, the 50th percentile ranged from 36.05 to 40.06 mL·kg^−1^·min^−1^. In boys, the 50th percentile of $\dot{V}$O_2_peak ranged from 44.66 to 47.08 mL·kg^−1^·min^−1^. In both boys and girls, there were higher levels of performance across all age groups, with most apparent gains between the ages of 13 and 14 years. However, from all ages $\dot{V}$O_2_peak was significantly lower in girls. 

**Figure 1:**
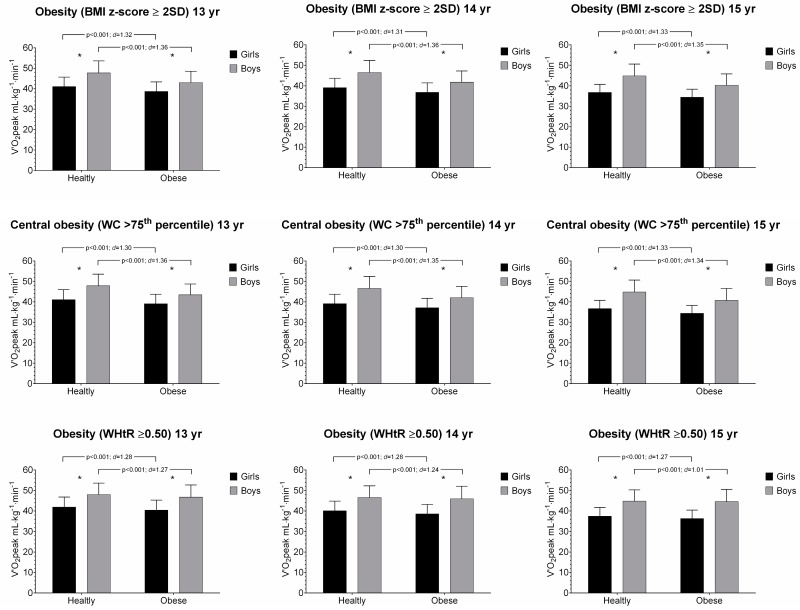
Relative peak oxygen uptake ($\dot{V}$O_2_peak) and anthropometric parameters (BMI z-score, waist circumference (WC) and waist-to-height ratio (WHtR)) by age and sex group. Values are mean and standard deviation (SD). * *p* < 0.001 for sex.

**Figure 2:**
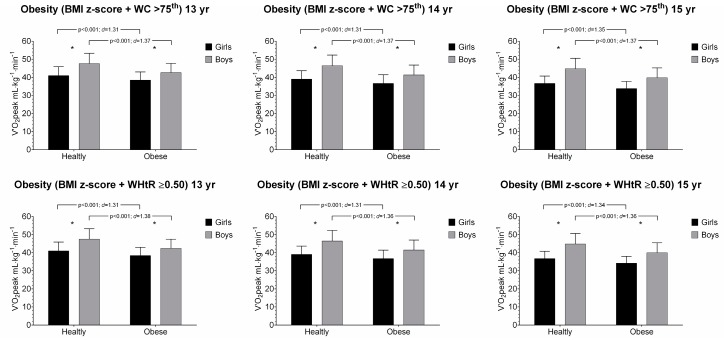
Relative peak oxygen uptake ($\dot{V}$O_2_peak) and combined anthropometric parameters (BMI z-score + WC and BMI z-score + WHtR) by age and sex group. Values are mean and standard deviation. * *p* < 0.001 for sex.

The associations between $\dot{V}$O_2_peak and anthropometric parameters in girls and boys aged 13 to 15 years old are presented in Figure 1 and Figure 2. Across all age and sex groups, participants categorized in healthy group had shown a significantly higher performance than their obese counterparts in $\dot{V}$O_2_peak. Overall, the healthy group had also higher CRF levels than obese counterparts using combined fatness parameters (i.e., BMI z-score + WC, and BMI z-score + WHtR), Figure 2.

## 4. Discussion

The present study is the first and largest study to provide age- and sex percentiles for CRF on south American adolescents aged 13–15 years old. The results of our study show that boys consistently outperformed girls in every age. In addition, the anthropometric parameters were inversely associated with CRF in all ages in both sexes. Overall, the obese group (by combined anthropometric parameters) had lower CRF levels than their non-obese counterparts.

Studies have shown CRF as a relevant marker of current and future health from childhood [10,27], highlighting the need for meaningful and accurate CRF assessment in early ages. However, since adolescence has been reported as period of life of chronological development and several metabolic changes (i.e., maturity status, body size, and body composition) [28], the correct interpretation of CRF assessment requires comparing data obtained in a particular person with normative values of that specific (general) population in the same age and sex [13]. In this line of thought, our study, provides contemporary reference data on sex- and age specific normative values for CRF in Latin America adolescents aged 13–15 years old. These values have utility for health and fitness screening, profiling, monitoring, and surveillance among Latin American Adolescents in this age group. 

Recently, a systematic review [13] was undertaken to identify studies explicitly reporting descriptive 20mSRT data on children and youth since 1981. The final data set included 1,142,026 subjects from 50 countries, extracted from 177 studies. In our study we presented $\dot{V}$O_2_peak centiles by age and sex, similar to those presented for Tomkinson et al [13]. For example, in the current study, the 50th percentile values of CRF ($\dot{V}$O_2_peak, mL·kg^−1^·min^−1^) for 13–15 years old adolescents, were 40.0, 38.0, 36.0 mL·kg^−1^·min^−1^ for girls, and 47.0, 45.8, 44.6 mL·kg^−1^·min^−1^ for boys, respectively, while in the study of Tomkinson et al. [13] for girls were 40.4, 38.8, 37.2 and for boys 45.0, 44.6, 44.0 mL·kg^−1^·min^−1^. It is not clear why such differences exist, but factors such as local physical activity levels, screen time, nutritional outcomes, body composition, stage maturation, or administration protocols (i.e., weather, terrain, and motivation for maximal effort for the entire test duration), could partially explain these differences. However, the influence of these factors remains speculative and should be investigated further. In this line, our findings extend these previous analyses by specifically using a large data sample of Latin America adolescents.

In the last decade, the CRF status of adolescents has been a concern [10,11]. A large part of this concern is based on the increase of prevalence of metabolic risk factors [29] and the obesity epidemic in children and adolescents [15,29]. Indeed, the CRF levels has declined over the last few decades, which seems to be associated to the current problems of sedentary lifestyle, overweight, and obesity [11]. As suggested by previous research [16,30], our results showed consistent and inversely associations across ages and sex groups between anthropometric parameters and CRF. Moreover, we also tested the influence of anthropometric parameters on the CRF across age groups, with a focus on obese and healthy groups and we found significant differences on $\dot{V}$O_2_peak, mL·kg^−1^·min^−1^ between groups. Overall, the healthy group had higher CRF levels than obese counterparts in both sexes using combined anthropometrics. These findings are in agreement with previous literature [11,16,31], where obese adolescents have been reported to have decreased physical activity and consequently reduced CRF levels. 

Since low CRF is associated with high cardiometabolic risk [9,10] and with tracking into adulthood [10] as well as obese adolescents being around five times more likely to be obese in adulthood than those who were not obese [32], our findings provide further support to implement intervention programs to reduce/prevent obesity in youth and to promote physical activity/exercise as a means to improve CRF levels. It is important to recognize that CRF in youth can be improved on average by 8–9% in response to an appropriate 12-week training programme independent of sex, age and maturation [33]. In addition, as 20mSRT performance is favourably associated with aspects of physiological, physical, psychosocial and cognitive health, due to its low cost and flexibility, our specific percentile values for $\dot{V}$O_2_peak, mL·kg^−1^·min^−1^ can be used to assess the CRF status within a country, region, community, and to help identify youth at risk of poor health. This form of monitoring is valuable both for the design and targeting of health and interventions to increase CRF levels as well as to reduce rates of obesity, particularly among Latin America youth populations.

The main limitation in this study is related to its design. Unfortunately, there is no data on pubertal stage, diet intake, physical activity, socio-economic background, habits, family medical records data, or cardiovascular risk factors (i.e., blood pressure, blood-based biomarkers, body composition, etc.) and this warrants further investigation in future studies. It should also be recognized that the studied sample is not representative of the Chilean or Colombian adolescent population. The strengths of this study included the potential utility of the sex- and age-specific normative values for $\dot{V}$O_2_peak that is important for health and fitness screening, profiling, monitoring, and surveillance in Latin American adolescents. In addition, the tests were conducted on a large sample of Latin American adolescents aged 13–15 years old and collected with standardized protocols. 

## 5. Conclusions

In summary, our study provides age- and sex-specific reference values for CRF ($\dot{V}$O_2_peak, mL·kg^−1^·min^−1^) on a large sample of Latin American adolescents from Chile and Colombia. As the 20mSRT is a simple, low cost method of estimation CRF its use is urged at a population level, whilst using the CRF percentiles to allow stratification and effective identification of individuals in need of physical activity and fitness promotion. Additionally, these data of adolescents aged 13–15 years complement the study published by Prieto-Benavides et al. [34] in the use of the Receiver Operating Characteristic analysis cut points to predict body adiposity parameters in practice by general practitioners, teachers, and coaches, may ensure that adolescents can be referred into intervention services effectively using a CRF assessment in a school setting. 

## Figures and Tables

**Table 1:** Characteristics of the sample by age and sex group.

Characteristics	*N*	Full Sample	Girls (*n* = 34,461)			Boys (*n* = 38,044)		
			13 Years Old (*n* = 8088)	14 Years Old (*n* = 20,108)	15 Years Old (*n* = 9848)	13 Years Old (*n* = 8025)	14 Years Old (*n* = 18,414)	15 Years Old (*n* = 8022)
Weight (kg)	72,419	56.01 (10.30)	54.62 (9.95) *	54.82 (9.60) *	54.26 (8.87) *	56.35 (11.08)	57.34 (10.92)	57.78 (10.34)
Height (cm)	72,411	1.61 (0.08)	1.56 (0.06) *	1.57 (0.06) *	1.57 (0.06) *	1.62 (0.08)	1.64 (0.07)	1.66 (0.07)
BMI (kg/m^2^)	72,393	21.65 (3.53)	22.29 (3.71) *	22.22 (3.53) *	22.12 (3.35) *	21.42 (3.59)	21.17 (3.49)	20.85 (3.18)
BMI z-score (WHO) ^a^	72,393	0.64 (1.04)	0.96 (0.97)	0.70 (0.96) *	0.47 (0.94) *	0.96 (1.04)	0.59 (1.07)	0.22 (1.05)
Obesity (≥2SD) ^a^ *N* (%)	72,305	6945 (9.6)	1106 (3.2) *	1582 (4.6) *	405 (1.2) *	1327 (3.5)	1973 (5.2)	552 (1.5)
Waist circumference (cm)	72,335	71.12 (8.48)	70.46 (8.62) *	70.12 (8.38) *	69.39 (7.70) *	72.74 (9.08)	72.18 (8.63)	71.47 (7.80)
Central obesity (>75th percentile for age and sex) ^a,b^ *N* (%)	72,335	8088 (11.2)	1329 (3.9) *	1859 (5.4) *	411 (1.2) *	1630 (4.3)	2241 (5.9)	618 (1.6)
Waist-to-height ratio	72,289	0.44 (0.05)	0.45 (0.05)	0.45 (0.05)	0.44 (0.05)	0.45 (0.05)	0.44 (0.05)	0.43 (0.05)
Obesity (≥0.50) ^a^ *N* (%)	72,289	10,897 (15.0)	1543 (4.5) *	3063 (8.9) *	1127 (3.3) *	1512 (4.0)	2747 (7.2)	905 (2.4)
BMI and waist circumference ^a^ *N* (%)	72,180	4971 (6.9)	801 (2.3) *	1068 (3.1) *	225 (0.7) *	1041 (2.7)	1451 (3.8)	385 (1.0)
BMI and waist-to-height ratio ^a^ *N* (%)	72,162	5528 (7.6)	848 (2.5)	1311 (3.8) *	345 (1.0)	993 (2.6)	1579 (4.2)	452 (1.2)
$\dot{V}$O_2_peak (mL·kg^−1^·min^−1^)–Léger et al.	72,505	42.49 (6.53)	40.72 (4.95) *	38.90 (4.67) *	36.60 (4.10) *	46.93 (5.89)	46.04 (6.06)	44.56 (5.91)

Data in mean and SD or frequency and percentage ^a^; >75th percentile waist circumference definition established by De Ferranti et al. [22] ^b^. BMI: body mass index; * *p* < 0.001 for comparison between sexes.

**Table 2:** Association between $\dot{V}$O_2_peak (20-meter shuttle run test) according to equation of Léger et al. and fatness parameters by age and sex group.

Fatness Parameters	Sex Group					
	Girls			Boys		
	13 Years Old	14 Years Old	15 Years Old	13 Years Old	14 Years Old	15 Years Old
BMI (kg/m^2^)	−0.21 (−0.23 to −0.19)	−0.22 (−0.23 to −0.21)	−0.19 (−0.21 to −0.17)	−0.31 (−0.33 to −0.29)	−0.27 (−0.28 to −0.25)	−0.22 (−0.24 to −0.20)
WC (cm)	−0.18 (−0.20 to −0.16)	−0.19 (−0.21 to −0.17)	−0.19 (−0.22 to −0.17)	−0.31 (−0.32 to −0.29)	−0.25 (−0.27 to −0.24)	−0.24 (−0.26 to −0.22)
WHtR	−0.18 (−0.20 to −0.16)	−0.20 (−0.21 to −0.18)	−0.21 (−0.23 to −0.19)	−0.34 (−0.36 to −0.32)	−0.29 (−0.30 to −0.27)	−0.26 (−0.28 to −0.24)

BMI: body mass index; WC: waist circumference; All *p* values are <0.001. Data in standardized regression coefficient with (95% confidence interval). Analysis was adjusted by country.

**Table 3:** Smooth centile scores and Cole’s transformation (L for skewness), median (M), and coefficient of variation (S) values of $\dot{V}$O_2_peak (20-meter shuttle run test) according to equation of Léger et al. by age and sex group.

	*N*	Mean	SD	L	S	3rd	10th	25th	50th (M)	75th	90th	97th
**Girls**												
13 years old	8025	40.72	4.94	−1.95	0.11	33.34	35.20	37.40	40.06	43.37	47.65	53.47
14 years old	18,414	38.89	4.66	−2.00	0.10	31.87	33.58	35.61	38.06	41.09	44.99	50.26
15 years old	8022	36.59	4.09	−2.05	0.10	30.37	31.95	33.81	36.05	38.82	42.36	47.11
**Boys**												
13 years old	8088	46.92	5.89	0.98	0.12	35.15	39.12	43.10	47.08	51.07	55.06	59.06
14 years old	20,108	46.03	6.05	0.94	0.13	33.99	37.93	41.89	45.88	49.88	53.91	57.94
15 years old	9848	44.56	5.90	0.91	0.13	32.84	36.74	40.68	44.66	48.66	52.70	56.76
